# [(*E*)-Oxido(pyridin-2-yl­methyl­idene)amine-κ^2^ *N*,*N*′][(*E*)-*N*-(pyridin-2-yl­methyl­idene)hydroxyl­amine-κ^2^ *N*,*N*′]silver(I) perchlorate–bis­[(*E*)-*N*-(pyridin-2-yl­methyl­idene)hydroxyl­amine-κ^2^ *N*,*N*′]silver(I) (1/1)

**DOI:** 10.1107/S160053681201625X

**Published:** 2012-04-21

**Authors:** Jing Xu, Shan Gao, Seik Weng Ng, Edward R. T. Tiekink

**Affiliations:** a Key Laboratory of Functional Inorganic Material Chemistry, Ministry of Education, Heilongjiang University, Harbin 150080, People’s Republic of China; b Department of Chemistry, University of Malaya, 50603 Kuala Lumpur, Malaysia; c Chemistry Department, Faculty of Science, King Abdulaziz University, PO Box 80203 Jeddah, Saudi Arabia

## Abstract

In the title salt co-crystal, [Ag(C_6_H_5_N_2_O)(C_6_H_6_N_2_O)]ClO_4_·[Ag(C_6_H_6_N_2_O)_2_], the asymmetric unit comprises a [Ag(*L*H)_2_]^+^ cation, a perchlorate anion and a neutral (*L*H)Ag*L* mol­ecule, where *L*H is pyridine-2-carboxaldoxime. Both silver-containing species feature *N*,*N*′-chelating *L*H and *L* ligands, which define an N_4_ donor set that is highly distorted [dihedral angles between AgC_2_N_2_ chelate rings = 45.7 (3) and 44.3 (2)°, respectively] owing, in part, to the close approach of a neighbouring Ag atom, leading to an argentophilic inter­action [Ag⋯Ag = 3.1868 (11) Å]. The mol­ecular conformations are stabilized by intra­molecular O—H⋯O hydrogen bonds. In the crystal, O—H⋯O inter­actions lead to supra­molecular chains along [010]. Chains aggregate into layers in the *ab* plane, defining channels along [100] in which reside the perchlorate anions; the latter are disordered over two overlapped orientations in a 50:50 ratio.

## Related literature

For structural diversity in the structures of silver salts, see: Kundu *et al.* (2010 ▶). For a related structure, see: Abu-Youssef *et al.* (2010 ▶).
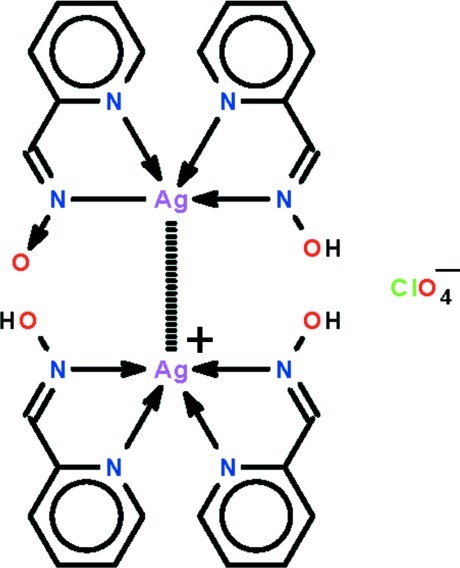


## Experimental

### 

#### Crystal data

[Ag(C_6_H_5_N_2_O)(C_6_H_6_N_2_O)]ClO_4_·[Ag(C_6_H_6_N_2_O)_2_]
*M* *_r_* = 802.69
Triclinic, 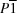

*a* = 7.3925 (18) Å
*b* = 8.3419 (19) Å
*c* = 25.626 (6) Å
α = 90.226 (6)°
β = 92.753 (6)°
γ = 114.409 (6)°
*V* = 1436.9 (6) Å^3^
*Z* = 2
Mo *K*α radiation
μ = 1.52 mm^−1^
*T* = 293 K
0.21 × 0.13 × 0.13 mm

#### Data collection

Rigaku R-AXIS RAPID IP diffractometer
Absorption correction: multi-scan (*ABSCOR*; Higashi, 1995 ▶) *T*_min_ = 0.356, *T*_max_ = 1.000
11387 measured reflections
5042 independent reflections
3660 reflections with *I* > 2σ(*I*)
*R*_int_ = 0.046

#### Refinement

*R*[*F* ^2^ > 2σ(*F* ^2^)] = 0.059
*wR*(*F* ^2^) = 0.187
*S* = 1.09
5042 reflections
430 parameters
64 restraints
H-atom parameters constrained
Δρ_max_ = 1.19 e Å^−3^
Δρ_min_ = −0.58 e Å^−3^

### 

Data collection: *RAPID-AUTO* (Rigaku, 1998 ▶); cell refinement: *RAPID-AUTO*; data reduction: *CrystalClear* (Rigaku/MSC and Rigaku, 2002 ▶); program(s) used to solve structure: *SHELXS97* (Sheldrick, 2008 ▶); program(s) used to refine structure: *SHELXL97* (Sheldrick, 2008 ▶); molecular graphics: *X-SEED* (Barbour, 2001 ▶) and *DIAMOND* (Brandenburg, 2006 ▶); software used to prepare material for publication: *publCIF* (Westrip, 2010 ▶).

## Supplementary Material

Crystal structure: contains datablock(s) global, I. DOI: 10.1107/S160053681201625X/hb6736sup1.cif

Structure factors: contains datablock(s) I. DOI: 10.1107/S160053681201625X/hb6736Isup2.hkl

Additional supplementary materials:  crystallographic information; 3D view; checkCIF report

## Figures and Tables

**Table 1:** Selected bond lengths (Å)

Ag1—N1	2.280 (5)
Ag1—N2	2.392 (5)
Ag1—N3	2.281 (5)
Ag1—N4	2.384 (5)
Ag2—N5	2.235 (5)
Ag2—N6	2.448 (4)
Ag2—N7	2.256 (5)
Ag2—N8	2.401 (5)

**Table 2:** Hydrogen-bond geometry (Å, °)

*D*—H⋯*A*	*D*—H	H⋯*A*	*D*⋯*A*	*D*—H⋯*A*
O1—H1*o*⋯O4	0.84	1.91	2.673 (8)	151
O3—H3*o*⋯O2	0.84	1.81	2.610 (6)	160
O4—H4*o*⋯O2^i^	0.84	1.64	2.475 (6)	174

Symmetry code: (i) 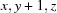.

**Fig. 1.**
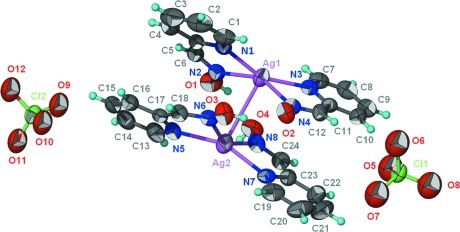
The molecular structure of (I) showing displacement ellipsoids at the 20% probability level. Each perchlorate anion has half-weight and for each, only one orientation is shown.

**Fig. 2.**
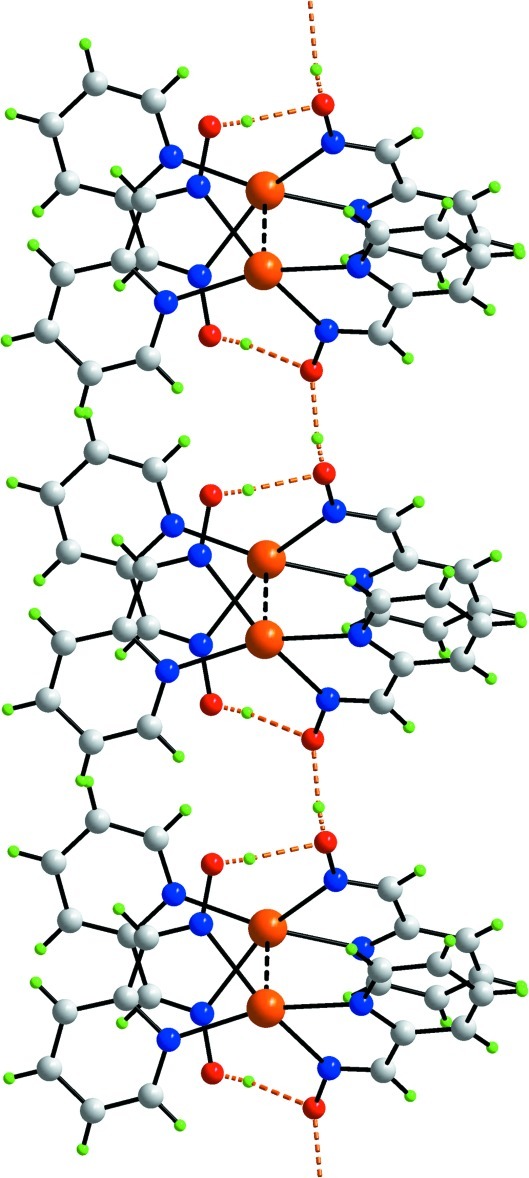
A view of the supramolecular chain along [010] in (I). The O—H···O hydrogen bonds are shown as orange dashed lines.

**Fig. 3.**
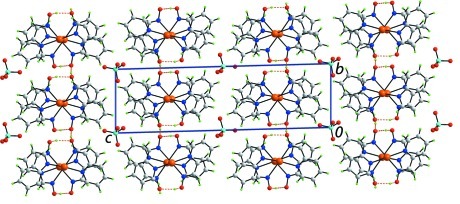
A view in projection down the a axis of the unit-cell contents for (I). The O—H···O interactions are shown as orange dashed lines. Only one orientation of the disordered perchlorate anions is shown.

## supplementary crystallographic information

### Comment

Nitrogen adducts of silver salts are notorious for their structural diversity with very different coordination geometries let alone supramolecular architectures being observed by a simple change in counter-ion, for example (Kundu *et al.*, 2010). A molecule that has yet to attract significant interest in terms of coordination chemistry towards silver is pyridine-2-carboxaldoxime (LH) with only the Ag(LH)NO_3_ salt reported thus far (Abu-Youssef *et al.*, 2010). Herein, the crystal structure determination of the perchlorate analogue (I) is described.

In (I), the asymmetric unit comprises a neutral (LH)AgL molecule, a [Ag(LH)_2_]^+^ cation and a perchlorate anion, Fig. 1. In both silver-containing species, the pyridine-2-carboxaldoxime and the derived anion are *N*,*N*-chelating, Table 1. The Ag—*N*(pyridine) and Ag—N(oxime) bond lengths are systematically shorter and longer, respectively, in the neutral molecule than in the cation, Table 1, and the Ag—N4(oximate) distance is shorter compared with the Ag—N(oxime) bond lengths, Table 1. Each five-membered AgC_2_N_2_ chelate ring is essentially planar with r.m.s. deviations of 0.062, 0.049, 0.080 and 0.035 Å, respectively for the N1, N3, N4 and N6-containing rings. The dihedral between the chelate rings involving the Ag1 atom is 44.3 (2)° indicating a distorted coordination geometry; the comparable angle for the Ag2-containing molecule is 45.7 (3)°. The close approach of a neighbouring silver atom contributes to the distortion as the silver atoms are connected by an agentophilic Ag1···Ag2 interaction, Table 1.

The most prominent feature of the crystal packing is the formation of a supramolecular chain along [010] mediated by O—H···O interactions, Fig. 2 and Table 2. In both the neutral (LH)AgL molecule and in the [Ag(LH)_2_]^+^ cation an intramolecular O—H···O interaction is formed. Links between molecules to form the chain are also of the type O—H···O and involve the formally anionic O2 atom which is bifurcated. A detailed analysis of the crystal packing is precluded owing to the disorder associated with the perchlorate anions. However, globally the Ag-containing molecules aggregate into layers in the *ab* plane to define channels along [100] in which reside the perchlorate anions, Fig. 3.

### Experimental

Silver perchlorate (1 mmol) and picolinaldehyde oxime (1 mmol) was dissolved in methanol solution (10 ml). The solution was filtered and set aside, away from light, for the growth of crystals. Colourless prisms deposited after several days.

### Refinement

Carbon- and oxygen-bound H-atoms were placed in calculated positions (C—H = 0.93 and O—H = 0.84 Å) and were included in the refinement in the riding model approximation, with *U*(H) set to 1.2–1.5*U*(C,*O*).

The perchlorate anion is disordered over two sites of equal weight, with each of the two chlorides located on a centre of inversion. The oxygen atoms of each perchlorate anion are further disordered and assumed to have a 1:1 type of disorder. All Cl—O distances were restrained to 1.41±0.01 Å and the O···O distances to 2.30±0.01 Å. Each set of four oxygen atoms was restrained to have the same anisotropic displacement parameters and these were restrained to be nearly isotropic.

The final difference Fourier map had a peak at 0.91 Å from the Ag1 atom.

### Crystal data

**Table:**

[Ag(C_6_H_5_N_2_O)(C_6_H_6_N_2_O)]ClO_4_·[Ag(C_6_H_6_N_2_O)_2_]	*Z* = 2
*M**_r_* = 802.69	*F*(000) = 796
Triclinic, *P*1	*D*_x_ = 1.855 Mg m^−^^3^
Hall symbol: -P 1	Mo *K*α radiation, λ = 0.71073 Å
*a* = 7.3925 (18) Å	Cell parameters from 7282 reflections
*b* = 8.3419 (19) Å	θ = 3.0–27.4°
*c* = 25.626 (6) Å	µ = 1.52 mm^−^^1^
α = 90.226 (6)°	*T* = 293 K
β = 92.753 (6)°	Prism, colourless
γ = 114.409 (6)°	0.21 × 0.13 × 0.13 mm
*V* = 1436.9 (6) Å^3^	

### Data collection

**Table:**

Rigaku R-AXIS RAPID IP diffractometer	5042 independent reflections
Radiation source: fine-focus sealed tube	3660 reflections with *I* > 2σ(*I*)
Graphite monochromator	*R*_int_ = 0.046
ω scan	θ_max_ = 25.0°, θ_min_ = 3.0°
Absorption correction: multi-scan (*ABSCOR*; Higashi, 1995)	*h* = −7→8
*T*_min_ = 0.356, *T*_max_ = 1.000	*k* = −10→10
11387 measured reflections	*l* = −30→30

### Refinement

**Table:**

Refinement on *F*^2^	Primary atom site location: structure-invariant direct methods
Least-squares matrix: full	Secondary atom site location: difference Fourier map
*R*[*F*^2^ > 2σ(*F*^2^)] = 0.059	Hydrogen site location: inferred from neighbouring sites
*wR*(*F*^2^) = 0.187	H-atom parameters constrained
*S* = 1.09	*w* = 1/[σ^2^(*F*_o_^2^) + (0.0985*P*)^2^ + 1.259*P*] where *P* = (*F*_o_^2^ + 2*F*_c_^2^)/3
5042 reflections	(Δ/σ)_max_ = 0.001
430 parameters	Δρ_max_ = 1.19 e Å^−^^3^
64 restraints	Δρ_min_ = −0.58 e Å^−^^3^

### Fractional atomic coordinates and isotropic or equivalent isotropic displacement parameters (Å^2^)

**Table:**

	*x*	*y*	*z*	*U*_iso_*/*U*_eq_	Occ. (<1)
Ag1	0.70514 (8)	0.49157 (7)	0.260394 (19)	0.0752 (2)	
Ag2	0.27551 (9)	0.48831 (8)	0.23871 (2)	0.0791 (2)	
Cl1	0.5000	0.0000	0.0000	0.0665 (6)	
Cl2	0.0000	1.0000	0.5000	0.0662 (6)	
O5	0.580 (3)	0.1840 (12)	0.0097 (9)	0.135 (5)	0.25
O6	0.597 (3)	−0.072 (3)	0.0356 (8)	0.135 (5)	0.25
O7	0.2954 (14)	−0.073 (3)	0.0089 (9)	0.135 (5)	0.25
O8	0.530 (4)	−0.037 (3)	−0.0512 (5)	0.135 (5)	0.25
O9	0.170 (2)	1.086 (2)	0.4738 (7)	0.110 (4)	0.25
O10	−0.089 (3)	0.8170 (12)	0.4851 (7)	0.110 (4)	0.25
O11	−0.143 (2)	1.069 (3)	0.4902 (8)	0.110 (4)	0.25
O12	0.043 (3)	1.008 (3)	0.5550 (3)	0.110 (4)	0.25
O5'	0.623 (3)	0.1818 (14)	−0.0045 (10)	0.151 (6)	0.25
O6'	0.433 (4)	−0.034 (3)	0.0503 (5)	0.151 (6)	0.25
O7'	0.336 (3)	−0.046 (4)	−0.0369 (8)	0.151 (6)	0.25
O8'	0.610 (3)	−0.097 (3)	−0.0124 (10)	0.151 (6)	0.25
O9'	0.040 (4)	1.033 (4)	0.4463 (4)	0.148 (6)	0.25
O10'	−0.136 (3)	0.8217 (16)	0.5034 (10)	0.148 (6)	0.25
O11'	−0.085 (4)	1.111 (3)	0.5179 (10)	0.148 (6)	0.25
O12'	0.178 (2)	1.031 (4)	0.5285 (10)	0.148 (6)	0.25
O1	0.7001 (8)	0.8834 (6)	0.3068 (2)	0.0852 (14)	
H1o	0.7215	0.9063	0.2752	0.128*	
O2	0.4335 (6)	0.0784 (5)	0.20609 (19)	0.0714 (12)	
O3	0.2203 (8)	0.0720 (5)	0.2850 (2)	0.0771 (13)	
H3o	0.2629	0.0680	0.2554	0.116*	
O4	0.6262 (7)	0.8966 (5)	0.2041 (2)	0.0786 (13)	
H4o	0.5628	0.9593	0.2071	0.118*	
N1	0.6765 (7)	0.3991 (6)	0.3444 (2)	0.0591 (12)	
N2	0.6812 (7)	0.7126 (6)	0.3159 (2)	0.0591 (12)	
N3	0.8625 (7)	0.5765 (6)	0.18431 (19)	0.0549 (11)	
N4	0.5644 (7)	0.2521 (5)	0.1986 (2)	0.0553 (11)	
N5	0.1855 (7)	0.5423 (6)	0.3166 (2)	0.0574 (12)	
N6	0.2256 (7)	0.2365 (6)	0.2941 (2)	0.0557 (11)	
N7	0.2272 (7)	0.4018 (6)	0.1538 (2)	0.0590 (12)	
N8	0.4962 (7)	0.7274 (6)	0.1894 (2)	0.0624 (13)	
C1	0.6511 (10)	0.2356 (9)	0.3590 (3)	0.0764 (19)	
H1A	0.6668	0.1610	0.3343	0.092*	
C2	0.6039 (12)	0.1754 (12)	0.4076 (4)	0.102 (3)	
H2	0.5821	0.0603	0.4156	0.122*	
C3	0.5889 (12)	0.2856 (14)	0.4448 (4)	0.104 (3)	
H3A	0.5595	0.2472	0.4786	0.125*	
C4	0.6173 (11)	0.4529 (13)	0.4322 (3)	0.088 (2)	
H4A	0.6096	0.5302	0.4573	0.106*	
C5	0.6581 (8)	0.5058 (9)	0.3807 (3)	0.0591 (15)	
C6	0.6811 (9)	0.6802 (8)	0.3638 (3)	0.0659 (16)	
H6	0.6957	0.7673	0.3885	0.079*	
C7	0.9996 (9)	0.7365 (8)	0.1739 (3)	0.0677 (17)	
H7	1.0476	0.8197	0.2012	0.081*	
C8	1.0742 (10)	0.7860 (9)	0.1258 (3)	0.0757 (19)	
H8	1.1715	0.8984	0.1208	0.091*	
C9	1.0014 (10)	0.6654 (10)	0.0853 (3)	0.0756 (19)	
H9	1.0489	0.6943	0.0521	0.091*	
C10	0.8557 (10)	0.4994 (10)	0.0943 (3)	0.0698 (17)	
H10	0.8016	0.4169	0.0670	0.084*	
C11	0.7920 (8)	0.4581 (7)	0.1443 (2)	0.0521 (13)	
C12	0.6440 (8)	0.2828 (8)	0.1552 (2)	0.0579 (14)	
H12	0.6081	0.1930	0.1299	0.070*	
C13	0.1915 (10)	0.6977 (9)	0.3323 (3)	0.0738 (18)	
H13	0.2120	0.7833	0.3074	0.089*	
C14	0.1695 (10)	0.7387 (9)	0.3823 (3)	0.0776 (19)	
H14	0.1757	0.8492	0.3911	0.093*	
C15	0.1382 (10)	0.6140 (11)	0.4194 (3)	0.078 (2)	
H15	0.1225	0.6385	0.4539	0.093*	
C16	0.1301 (10)	0.4511 (10)	0.4051 (3)	0.0703 (18)	
H16	0.1077	0.3644	0.4298	0.084*	
C17	0.1556 (7)	0.4185 (7)	0.3538 (2)	0.0509 (13)	
C18	0.1551 (9)	0.2518 (7)	0.3372 (2)	0.0577 (14)	
H18	0.1026	0.1548	0.3585	0.069*	
C19	0.1015 (12)	0.2427 (9)	0.1338 (3)	0.083 (2)	
H19	0.0159	0.1619	0.1560	0.100*	
C20	0.0939 (16)	0.1945 (12)	0.0825 (4)	0.109 (3)	
H20	0.0058	0.0828	0.0704	0.130*	
C21	0.2162 (19)	0.3111 (16)	0.0493 (4)	0.116 (3)	
H21	0.2131	0.2792	0.0143	0.139*	
C22	0.3443 (13)	0.4759 (12)	0.0674 (3)	0.086 (2)	
H22	0.4271	0.5580	0.0449	0.103*	
C23	0.3474 (11)	0.5172 (9)	0.1202 (3)	0.0662 (17)	
C24	0.4803 (10)	0.6905 (8)	0.1411 (3)	0.0661 (16)	
H24	0.5555	0.7762	0.1183	0.079*	

### Atomic displacement parameters (Å^2^)

**Table:**

	*U*^11^	*U*^22^	*U*^33^	*U*^12^	*U*^13^	*U*^23^
Ag1	0.0860 (4)	0.0851 (4)	0.0572 (4)	0.0386 (3)	0.0006 (3)	−0.0154 (3)
Ag2	0.0911 (4)	0.0918 (4)	0.0588 (4)	0.0408 (3)	0.0170 (3)	0.0185 (3)
Cl1	0.0764 (13)	0.0598 (12)	0.0535 (11)	0.0180 (10)	0.0091 (10)	−0.0010 (9)
Cl2	0.0880 (15)	0.0592 (12)	0.0552 (12)	0.0340 (11)	0.0075 (11)	0.0040 (9)
O5	0.145 (8)	0.127 (8)	0.126 (8)	0.046 (6)	0.015 (7)	−0.004 (7)
O6	0.145 (8)	0.127 (8)	0.126 (8)	0.046 (6)	0.015 (7)	−0.004 (7)
O7	0.145 (8)	0.127 (8)	0.126 (8)	0.046 (6)	0.015 (7)	−0.004 (7)
O8	0.145 (8)	0.127 (8)	0.126 (8)	0.046 (6)	0.015 (7)	−0.004 (7)
O9	0.124 (7)	0.113 (7)	0.091 (7)	0.048 (6)	0.015 (6)	−0.018 (6)
O10	0.124 (7)	0.113 (7)	0.091 (7)	0.048 (6)	0.015 (6)	−0.018 (6)
O11	0.124 (7)	0.113 (7)	0.091 (7)	0.048 (6)	0.015 (6)	−0.018 (6)
O12	0.124 (7)	0.113 (7)	0.091 (7)	0.048 (6)	0.015 (6)	−0.018 (6)
O5'	0.169 (9)	0.133 (9)	0.145 (10)	0.054 (8)	0.028 (8)	−0.012 (7)
O6'	0.169 (9)	0.133 (9)	0.145 (10)	0.054 (8)	0.028 (8)	−0.012 (7)
O7'	0.169 (9)	0.133 (9)	0.145 (10)	0.054 (8)	0.028 (8)	−0.012 (7)
O8'	0.169 (9)	0.133 (9)	0.145 (10)	0.054 (8)	0.028 (8)	−0.012 (7)
O9'	0.162 (9)	0.143 (9)	0.135 (9)	0.060 (8)	−0.001 (8)	−0.023 (7)
O10'	0.162 (9)	0.143 (9)	0.135 (9)	0.060 (8)	−0.001 (8)	−0.023 (7)
O11'	0.162 (9)	0.143 (9)	0.135 (9)	0.060 (8)	−0.001 (8)	−0.023 (7)
O12'	0.162 (9)	0.143 (9)	0.135 (9)	0.060 (8)	−0.001 (8)	−0.023 (7)
O1	0.104 (4)	0.050 (2)	0.107 (4)	0.039 (2)	−0.013 (3)	−0.011 (2)
O2	0.071 (3)	0.043 (2)	0.097 (3)	0.0182 (18)	0.019 (2)	−0.001 (2)
O3	0.093 (3)	0.041 (2)	0.102 (4)	0.029 (2)	0.028 (3)	0.010 (2)
O4	0.075 (3)	0.051 (2)	0.111 (4)	0.028 (2)	−0.006 (3)	0.000 (2)
N1	0.051 (2)	0.058 (3)	0.067 (3)	0.022 (2)	−0.003 (2)	−0.008 (2)
N2	0.060 (3)	0.051 (3)	0.069 (3)	0.027 (2)	−0.002 (2)	−0.010 (2)
N3	0.053 (2)	0.043 (2)	0.068 (3)	0.021 (2)	−0.004 (2)	−0.002 (2)
N4	0.055 (2)	0.037 (2)	0.071 (3)	0.0168 (19)	−0.003 (2)	−0.005 (2)
N5	0.057 (3)	0.048 (3)	0.071 (3)	0.024 (2)	0.014 (2)	0.010 (2)
N6	0.055 (2)	0.042 (2)	0.071 (3)	0.0211 (19)	0.001 (2)	0.004 (2)
N7	0.063 (3)	0.055 (3)	0.065 (3)	0.031 (2)	−0.009 (2)	0.003 (2)
N8	0.064 (3)	0.045 (3)	0.081 (4)	0.023 (2)	0.015 (3)	0.010 (2)
C1	0.067 (4)	0.065 (4)	0.097 (5)	0.029 (3)	−0.018 (4)	0.000 (4)
C2	0.078 (5)	0.086 (6)	0.126 (8)	0.020 (4)	−0.006 (5)	0.032 (6)
C3	0.074 (5)	0.120 (7)	0.101 (7)	0.023 (5)	0.003 (5)	0.046 (6)
C4	0.066 (4)	0.124 (7)	0.074 (5)	0.039 (4)	0.004 (4)	0.001 (5)
C5	0.050 (3)	0.072 (4)	0.058 (4)	0.028 (3)	−0.001 (3)	−0.008 (3)
C6	0.063 (3)	0.068 (4)	0.072 (4)	0.034 (3)	−0.010 (3)	−0.027 (3)
C7	0.067 (4)	0.050 (3)	0.087 (5)	0.028 (3)	−0.014 (3)	−0.013 (3)
C8	0.064 (4)	0.061 (4)	0.099 (5)	0.022 (3)	0.012 (4)	0.027 (4)
C9	0.068 (4)	0.080 (5)	0.083 (5)	0.034 (3)	0.017 (4)	0.031 (4)
C10	0.066 (4)	0.084 (5)	0.060 (4)	0.032 (3)	−0.001 (3)	−0.001 (3)
C11	0.050 (3)	0.050 (3)	0.058 (3)	0.024 (2)	−0.003 (3)	−0.002 (3)
C12	0.058 (3)	0.054 (3)	0.062 (4)	0.023 (3)	0.003 (3)	−0.016 (3)
C13	0.069 (4)	0.061 (4)	0.098 (5)	0.033 (3)	0.016 (4)	0.007 (4)
C14	0.062 (4)	0.066 (4)	0.106 (6)	0.029 (3)	0.005 (4)	−0.019 (4)
C15	0.062 (4)	0.102 (6)	0.076 (5)	0.041 (4)	0.000 (3)	−0.020 (4)
C16	0.060 (3)	0.089 (5)	0.068 (4)	0.036 (3)	0.006 (3)	0.010 (4)
C17	0.043 (3)	0.056 (3)	0.053 (3)	0.020 (2)	0.003 (2)	0.011 (3)
C18	0.061 (3)	0.049 (3)	0.065 (4)	0.025 (3)	0.013 (3)	0.019 (3)
C19	0.093 (5)	0.056 (4)	0.107 (6)	0.041 (4)	−0.020 (4)	−0.001 (4)
C20	0.143 (8)	0.089 (6)	0.110 (7)	0.072 (6)	−0.061 (6)	−0.040 (5)
C21	0.175 (10)	0.140 (9)	0.078 (6)	0.116 (8)	−0.038 (6)	−0.032 (6)
C22	0.102 (6)	0.110 (6)	0.065 (4)	0.064 (5)	−0.003 (4)	0.000 (4)
C23	0.081 (4)	0.080 (4)	0.059 (4)	0.054 (4)	0.006 (3)	0.012 (3)
C24	0.068 (4)	0.064 (4)	0.067 (4)	0.026 (3)	0.010 (3)	0.022 (3)

### Geometric parameters (Å, º)

**Table:**

Ag1—N1	2.280 (5)	N5—C13	1.338 (8)
Ag1—N2	2.392 (5)	N5—C17	1.365 (7)
Ag1—N3	2.281 (5)	N6—C18	1.274 (7)
Ag1—N4	2.384 (5)	N7—C19	1.348 (8)
Ag2—N5	2.235 (5)	N7—C23	1.353 (8)
Ag2—N6	2.448 (4)	N8—C24	1.263 (8)
Ag2—N7	2.256 (5)	C1—C2	1.352 (11)
Ag2—N8	2.401 (5)	C1—H1A	0.9300
Ag1—Ag2	3.1868 (11)	C2—C3	1.360 (13)
Cl1—O6'	1.392 (8)	C2—H2	0.9300
Cl1—O6'^i^	1.392 (8)	C3—C4	1.365 (12)
Cl1—O8^i^	1.395 (8)	C3—H3A	0.9300
Cl1—O8	1.395 (8)	C4—C5	1.399 (10)
Cl1—O8'	1.407 (9)	C4—H4A	0.9300
Cl1—O8'^i^	1.407 (9)	C5—C6	1.464 (9)
Cl1—O7	1.409 (8)	C6—H6	0.9300
Cl1—O7^i^	1.409 (8)	C7—C8	1.371 (10)
Cl1—O5	1.414 (8)	C7—H7	0.9300
Cl1—O5^i^	1.414 (8)	C8—C9	1.366 (10)
Cl1—O7'^i^	1.418 (9)	C8—H8	0.9300
Cl1—O7'	1.418 (9)	C9—C10	1.387 (10)
Cl2—O9	1.375 (8)	C9—H9	0.9300
Cl2—O9^ii^	1.375 (8)	C10—C11	1.382 (9)
Cl2—O11'^ii^	1.400 (9)	C10—H10	0.9300
Cl2—O11'	1.400 (9)	C11—C12	1.458 (8)
Cl2—O12'	1.400 (9)	C12—H12	0.9300
Cl2—O12'^ii^	1.400 (9)	C13—C14	1.359 (10)
Cl2—O11^ii^	1.410 (8)	C13—H13	0.9300
Cl2—O11	1.410 (8)	C14—C15	1.367 (10)
Cl2—O10'	1.415 (9)	C14—H14	0.9300
Cl2—O10'^ii^	1.415 (9)	C15—C16	1.383 (11)
Cl2—O12	1.424 (8)	C15—H15	0.9300
Cl2—O12^ii^	1.424 (8)	C16—C17	1.379 (9)
O1—N2	1.395 (6)	C16—H16	0.9300
O1—H1o	0.8400	C17—C18	1.450 (8)
O2—N4	1.391 (6)	C18—H18	0.9300
O3—N6	1.375 (6)	C19—C20	1.365 (12)
O3—H3o	0.8400	C19—H19	0.9300
O4—N8	1.378 (6)	C20—C21	1.357 (14)
O4—H4o	0.8400	C20—H20	0.9300
N1—C5	1.334 (8)	C21—C22	1.370 (13)
N1—C1	1.354 (8)	C21—H21	0.9300
N2—C6	1.257 (8)	C22—C23	1.392 (10)
N3—C7	1.338 (7)	C22—H22	0.9300
N3—C11	1.347 (7)	C23—C24	1.453 (10)
N4—C12	1.261 (7)	C24—H24	0.9300
			
N1—Ag1—N3	154.92 (17)	C2—C3—H3A	120.1
N1—Ag1—N4	112.19 (17)	C4—C3—H3A	120.1
N3—Ag1—N4	71.69 (16)	C3—C4—C5	118.7 (9)
N1—Ag1—N2	70.69 (18)	C3—C4—H4A	120.6
N3—Ag1—N2	118.35 (16)	C5—C4—H4A	120.6
N4—Ag1—N2	151.77 (17)	N1—C5—C4	121.6 (7)
N1—Ag1—Ag2	99.47 (12)	N1—C5—C6	116.6 (6)
N3—Ag1—Ag2	105.57 (12)	C4—C5—C6	121.8 (7)
N4—Ag1—Ag2	79.91 (11)	N2—C6—C5	120.1 (6)
N2—Ag1—Ag2	72.01 (12)	N2—C6—H6	119.9
N5—Ag2—N7	155.54 (18)	C5—C6—H6	119.9
N5—Ag2—N8	119.88 (17)	N3—C7—C8	124.4 (6)
N7—Ag2—N8	72.02 (18)	N3—C7—H7	117.8
N5—Ag2—N6	71.74 (16)	C8—C7—H7	117.8
N7—Ag2—N6	109.99 (17)	C9—C8—C7	118.0 (6)
N8—Ag2—N6	148.63 (16)	C9—C8—H8	121.0
N5—Ag2—Ag1	105.18 (13)	C7—C8—H8	121.0
N7—Ag2—Ag1	98.55 (12)	C8—C9—C10	119.3 (6)
N8—Ag2—Ag1	74.53 (12)	C8—C9—H9	120.4
N6—Ag2—Ag1	74.24 (11)	C10—C9—H9	120.4
O6'—Cl1—O8'	111.4 (8)	C11—C10—C9	119.1 (7)
O8—Cl1—O7	110.5 (8)	C11—C10—H10	120.4
O8—Cl1—O5	110.4 (8)	C9—C10—H10	120.4
O7—Cl1—O5	108.9 (8)	N3—C11—C10	121.9 (5)
O6'—Cl1—O7'	110.0 (8)	N3—C11—C12	117.8 (5)
O8'—Cl1—O7'	108.7 (8)	C10—C11—C12	120.3 (6)
O11'—Cl2—O12'	111.0 (8)	N4—C12—C11	120.4 (5)
O9—Cl2—O11	112.9 (8)	N4—C12—H12	119.8
O11'—Cl2—O10'	110.1 (8)	C11—C12—H12	119.8
O12'—Cl2—O10'	110.3 (8)	N5—C13—C14	124.4 (7)
O9—Cl2—O12	111.2 (8)	N5—C13—H13	117.8
N2—O1—H1o	109.5	C14—C13—H13	117.8
N6—O3—H3o	109.5	C13—C14—C15	118.6 (7)
N8—O4—H4o	109.5	C13—C14—H14	120.7
C5—N1—C1	117.5 (6)	C15—C14—H14	120.7
C5—N1—Ag1	117.3 (4)	C14—C15—C16	119.2 (7)
C1—N1—Ag1	124.6 (5)	C14—C15—H15	120.4
C6—N2—O1	112.4 (5)	C16—C15—H15	120.4
C6—N2—Ag1	114.2 (4)	C17—C16—C15	119.3 (7)
O1—N2—Ag1	132.4 (4)	C17—C16—H16	120.3
C7—N3—C11	117.2 (5)	C15—C16—H16	120.3
C7—N3—Ag1	126.8 (4)	N5—C17—C16	121.6 (6)
C11—N3—Ag1	115.6 (3)	N5—C17—C18	117.2 (5)
C12—N4—O2	115.8 (5)	C16—C17—C18	121.3 (5)
C12—N4—Ag1	113.9 (4)	N6—C18—C17	120.8 (5)
O2—N4—Ag1	129.4 (4)	N6—C18—H18	119.6
C13—N5—C17	116.9 (6)	C17—C18—H18	119.6
C13—N5—Ag2	124.8 (4)	N7—C19—C20	123.1 (8)
C17—N5—Ag2	117.3 (4)	N7—C19—H19	118.4
C18—N6—O3	113.6 (4)	C20—C19—H19	118.4
C18—N6—Ag2	111.1 (4)	C21—C20—C19	119.4 (8)
O3—N6—Ag2	133.8 (4)	C21—C20—H20	120.3
C19—N7—C23	116.9 (6)	C19—C20—H20	120.3
C19—N7—Ag2	126.6 (5)	C20—C21—C22	119.9 (9)
C23—N7—Ag2	116.3 (4)	C20—C21—H21	120.1
C24—N8—O4	115.8 (5)	C22—C21—H21	120.1
C24—N8—Ag2	112.5 (4)	C21—C22—C23	118.3 (9)
O4—N8—Ag2	131.6 (4)	C21—C22—H22	120.8
C2—C1—N1	123.3 (8)	C23—C22—H22	120.8
C2—C1—H1A	118.3	N7—C23—C22	122.4 (7)
N1—C1—H1A	118.3	N7—C23—C24	117.3 (6)
C1—C2—C3	119.0 (8)	C22—C23—C24	120.3 (7)
C1—C2—H2	120.5	N8—C24—C23	121.5 (6)
C3—C2—H2	120.5	N8—C24—H24	119.2
C2—C3—C4	119.8 (8)	C23—C24—H24	119.2
			
N1—Ag1—Ag2—N5	29.36 (18)	N6—Ag2—N7—C23	−143.4 (4)
N3—Ag1—Ag2—N5	−151.98 (17)	Ag1—Ag2—N7—C23	−67.1 (4)
N4—Ag1—Ag2—N5	140.41 (17)	N5—Ag2—N8—C24	−156.9 (4)
N2—Ag1—Ag2—N5	−36.65 (18)	N7—Ag2—N8—C24	−0.4 (4)
N1—Ag1—Ag2—N7	−144.67 (18)	N6—Ag2—N8—C24	98.7 (5)
N3—Ag1—Ag2—N7	34.00 (17)	Ag1—Ag2—N8—C24	104.2 (4)
N4—Ag1—Ag2—N7	−33.61 (17)	N5—Ag2—N8—O4	19.4 (5)
N2—Ag1—Ag2—N7	149.33 (17)	N7—Ag2—N8—O4	175.9 (5)
N1—Ag1—Ag2—N8	146.78 (19)	N6—Ag2—N8—O4	−85.0 (5)
N3—Ag1—Ag2—N8	−34.56 (18)	Ag1—Ag2—N8—O4	−79.5 (5)
N4—Ag1—Ag2—N8	−102.17 (18)	C5—N1—C1—C2	1.6 (9)
N2—Ag1—Ag2—N8	80.78 (19)	Ag1—N1—C1—C2	−169.4 (5)
N1—Ag1—Ag2—N6	−36.19 (17)	N1—C1—C2—C3	−2.8 (12)
N3—Ag1—Ag2—N6	142.47 (17)	C1—C2—C3—C4	1.4 (12)
N4—Ag1—Ag2—N6	74.86 (17)	C2—C3—C4—C5	1.0 (12)
N2—Ag1—Ag2—N6	−102.20 (18)	C1—N1—C5—C4	1.0 (8)
N3—Ag1—N1—C5	117.1 (5)	Ag1—N1—C5—C4	172.7 (5)
N4—Ag1—N1—C5	−148.8 (4)	C1—N1—C5—C6	−178.5 (5)
N2—Ag1—N1—C5	1.1 (4)	Ag1—N1—C5—C6	−6.8 (7)
Ag2—Ag1—N1—C5	−65.9 (4)	C3—C4—C5—N1	−2.3 (10)
N3—Ag1—N1—C1	−71.8 (6)	C3—C4—C5—C6	177.2 (7)
N4—Ag1—N1—C1	22.2 (5)	O1—N2—C6—C5	178.6 (5)
N2—Ag1—N1—C1	172.1 (5)	Ag1—N2—C6—C5	−11.2 (7)
Ag2—Ag1—N1—C1	105.1 (5)	N1—C5—C6—N2	12.6 (9)
N1—Ag1—N2—C6	5.5 (4)	C4—C5—C6—N2	−167.0 (6)
N3—Ag1—N2—C6	−148.9 (4)	C11—N3—C7—C8	−1.0 (9)
N4—Ag1—N2—C6	106.6 (5)	Ag1—N3—C7—C8	−172.7 (5)
Ag2—Ag1—N2—C6	112.8 (4)	N3—C7—C8—C9	1.3 (10)
N1—Ag1—N2—O1	173.1 (5)	C7—C8—C9—C10	0.3 (10)
N3—Ag1—N2—O1	18.8 (6)	C8—C9—C10—C11	−2.0 (10)
N4—Ag1—N2—O1	−85.7 (6)	C7—N3—C11—C10	−0.8 (8)
Ag2—Ag1—N2—O1	−79.6 (5)	Ag1—N3—C11—C10	171.8 (5)
N1—Ag1—N3—C7	−81.3 (6)	C7—N3—C11—C12	179.4 (5)
N4—Ag1—N3—C7	175.3 (5)	Ag1—N3—C11—C12	−8.0 (6)
N2—Ag1—N3—C7	24.1 (5)	C9—C10—C11—N3	2.3 (10)
Ag2—Ag1—N3—C7	101.8 (5)	C9—C10—C11—C12	−177.9 (6)
N1—Ag1—N3—C11	106.9 (5)	O2—N4—C12—C11	−176.6 (5)
N4—Ag1—N3—C11	3.5 (4)	Ag1—N4—C12—C11	−6.6 (7)
N2—Ag1—N3—C11	−147.7 (4)	N3—C11—C12—N4	10.1 (8)
Ag2—Ag1—N3—C11	−70.0 (4)	C10—C11—C12—N4	−169.6 (6)
N1—Ag1—N4—C12	−151.8 (4)	C17—N5—C13—C14	−0.1 (9)
N3—Ag1—N4—C12	1.7 (4)	Ag2—N5—C13—C14	−168.2 (5)
N2—Ag1—N4—C12	117.9 (5)	N5—C13—C14—C15	−0.3 (10)
Ag2—Ag1—N4—C12	112.0 (4)	C13—C14—C15—C16	0.1 (10)
N1—Ag1—N4—O2	16.5 (5)	C14—C15—C16—C17	0.6 (10)
N3—Ag1—N4—O2	170.0 (5)	C13—N5—C17—C16	0.9 (8)
N2—Ag1—N4—O2	−73.8 (6)	Ag2—N5—C17—C16	169.8 (4)
Ag2—Ag1—N4—O2	−79.7 (4)	C13—N5—C17—C18	−178.1 (5)
N7—Ag2—N5—C13	−91.9 (6)	Ag2—N5—C17—C18	−9.2 (6)
N8—Ag2—N5—C13	21.8 (6)	C15—C16—C17—N5	−1.1 (9)
N6—Ag2—N5—C13	169.8 (5)	C15—C16—C17—C18	177.9 (6)
Ag1—Ag2—N5—C13	102.5 (5)	O3—N6—C18—C17	177.9 (5)
N7—Ag2—N5—C17	100.0 (5)	Ag2—N6—C18—C17	−13.9 (7)
N8—Ag2—N5—C17	−146.2 (4)	N5—C17—C18—N6	16.3 (8)
N6—Ag2—N5—C17	1.7 (4)	C16—C17—C18—N6	−162.7 (6)
Ag1—Ag2—N5—C17	−65.6 (4)	C23—N7—C19—C20	1.1 (10)
N5—Ag2—N6—C18	6.4 (4)	Ag2—N7—C19—C20	−173.7 (6)
N7—Ag2—N6—C18	−147.7 (4)	N7—C19—C20—C21	−0.7 (12)
N8—Ag2—N6—C18	124.2 (4)	C19—C20—C21—C22	−0.6 (14)
Ag1—Ag2—N6—C18	118.7 (4)	C20—C21—C22—C23	1.3 (13)
N5—Ag2—N6—O3	171.5 (5)	C19—N7—C23—C22	−0.3 (9)
N7—Ag2—N6—O3	17.3 (5)	Ag2—N7—C23—C22	175.0 (5)
N8—Ag2—N6—O3	−70.7 (6)	C19—N7—C23—C24	178.7 (6)
Ag1—Ag2—N6—O3	−76.2 (5)	Ag2—N7—C23—C24	−6.0 (7)
N5—Ag2—N7—C19	−58.3 (7)	C21—C22—C23—N7	−0.9 (11)
N8—Ag2—N7—C19	178.2 (5)	C21—C22—C23—C24	−179.8 (7)
N6—Ag2—N7—C19	31.4 (5)	O4—N8—C24—C23	−179.6 (5)
Ag1—Ag2—N7—C19	107.6 (5)	Ag2—N8—C24—C23	−2.7 (8)
N5—Ag2—N7—C23	126.9 (5)	N7—C23—C24—N8	6.0 (9)
N8—Ag2—N7—C23	3.4 (4)	C22—C23—C24—N8	−175.0 (6)

Symmetry codes: (i) −*x*+1, −*y*, −*z*; (ii) −*x*, −*y*+2, −*z*+1.

### Hydrogen-bond geometry (Å, º)

**Table:**

*D*—H···*A*	*D*—H	H···*A*	*D*···*A*	*D*—H···*A*
O1—H1*o*···O4	0.84	1.91	2.673 (8)	151
O3—H3*o*···O2	0.84	1.81	2.610 (6)	160
O4—H4*o*···O2^iii^	0.84	1.64	2.475 (6)	174

Symmetry code: (iii) *x*, *y*+1, *z*.

## References

[bb1] Abu-Youssef, M. A. M., Soliman, S. M., Langer, V., Gohar, Y. M., Hasanen, A. A., Makhyoun, M. A., Zaky, A. H. & Ohrstrom, L. R. (2010). *Inorg. Chem* **49**, 9788–9797. 10.1021/ic100581k 20929250

[bb2] Barbour, L. J. (2001). *J. Supramol. Chem.* **1**, 189–191.

[bb3] Brandenburg, K. (2006). *DIAMOND* Crystal Impact GbR, Bonn, Germany.

[bb4] Higashi, T. (1995). *ABSCOR* Rigaku Corporation, Tokyo, Japan.

[bb5] Kundu, N., Audhya, A., Towsif Abtab, Sk. Md., Ghosh, S., Tiekink, E. R. T. & Chaudhury, M. (2010). *Cryst. Growth Des.* **10**, 1269–1282.

[bb6] Rigaku (1998). *RAPID-AUTO* Rigaku Corporation, Tokyo, Japan.

[bb7] Rigaku/MSC and Rigaku (2002). *CrystalClear* Rigaku/MSC Inc., The Woodlands, Texas, USA.

[bb8] Sheldrick, G. M. (2008). *Acta Cryst.* A**64**, 112–122. 10.1107/S0108767307043930 18156677

[bb9] Westrip, S. P. (2010). *J. Appl. Cryst.* **43**, 920–925.

